# Oral health knowledge is associated with oral health-related quality of life: a survey of first-year undergraduate students enrolled in an American university

**DOI:** 10.1186/s12903-023-03721-5

**Published:** 2023-12-03

**Authors:** Jenna Gardner, Boyen Huang, Ryan H. L. Ip

**Affiliations:** 1 School of Dentistry, University of Minnesota Twin Cities, 515 Delaware St SE, Minneapolis, MN 55455 USA; 2 School of Computing, Mathematics and Engineering, Charles Sturt University, Wagga Wagga, NSW Australia

**Keywords:** Oral health knowledge, Oral health-related quality of life (OHRQoL), Comprehensive measure of oral health knowledge (CMOHK), Undergraduate, Young adult, Ordinal regression

## Abstract

**Background:**

Oral health knowledge forms part of oral health literacy that enables individuals to inform appropriate oral health decisions and actions. Oral health-related quality of life (OHRQoL) characterizes self-perception of well-being influenced by oral health. This study aimed to examine the relationship between oral health knowledge and OHRQoL.

**Methods:**

A random sample of 19-to-24-year-old first-year undergraduate students (n = 372) in Minnesota, United States of America was used. Each student was assessed with an online survey using the Comprehensive Measure of Oral Health Knowledge (CMOHK) and the OHRQoL items of the World Health Organization (WHO) Oral Health Questionnaire for Adults. Relationships between OHRQoL parameters and CMOHK together with other covariates were assessed using ordinal regression models. Associations between OHRQoL parameters were examined with the Kendall’s tau-b method.

**Results:**

Dry mouth (45%) was the most reported OHRQoL issue. The respondents showing good oral health knowledge were less likely to experience speech or pronunciation difficulty (β=-1.12, *p* = 0.0006), interrupted sleep (β=-1.43, *p* = 0.0040), taking days off (β=-1.71, *p* = 0.0054), difficulty doing usual activities (β=-2.37, *p* = 0.0002), or reduced participation in social activities due to dental or oral issues (β=-1.65, *p* = 0.0078).

**Conclusions:**

This study suggested a protective effect of better oral health knowledge on specific OHRQoL issues. In addition to provision of affordable dental services, university-wide oral health education can be implemented to improve OHRQoL in undergraduate students.

## Background

Undergraduate education, or college in the United States (US) system, is commonly referred to as the first step to freedom in one’s life and for many, it is an opportunity to gain invaluable life experience. With new sense of independence comes the responsibilities of decision making. The absence of parental reminders to brush and floss before bed, to eat healthy, and to schedule a 6-month recall dental visit can lead to a lack of adherence of proper health practices. Further, in the US, dental care is costly, and not all children and young adults are covered by dental insurance [1]. Approximately 25% of undergraduate students missed the relevant annual dental checkup and cleaning, and the percentage increased from 15.2% in the freshman year to 24.4% in the fourth year of study [2]. Lack of adherence of appropriate oral health behavior such as toothbrushing, flossing, use of mouthwash, and regular dental visits was prevalent in young adults [3], which predisposed college freshmen to some oral diseases such as gingivitis [4].

In addition, an inadequate level of oral health knowledge (the information about the occurrence and recommended actions of specific oral health issues [5]) was prevalent among college students, which was also a determinant of unfavorable oral conditions in this population [6]. Oral health knowledge forms part of oral health literacy that is defined as the capacity level to enable individuals to make appropriate oral health informed decisions and actions [7–9]. In this regard, college students having better oral health knowledge would perform unhealthy oral health behavior less often [10].

While a relationship between college students’ oral health knowledge and oral conditions has been suggested, the association between college students’ oral health knowledge and oral health-related quality of life (OHRQoL) was barely investigated. OHRQoL represents an individual’s self-perceived well-being from the perspectives of oral function, orofacial pain, orofacial appearance, and psychosocial impact [11]. At the time the present study was planned, to the best of the authors’ knowledge, only a study in China has reported an association between undergraduate students’ oral health knowledge and OHRQoL [12]. There was a study in Saudi Arabia reporting this topic after we had completed data collection and analysis although their work was published earlier than ours [13]. As for the situations in the US, a California-based study has demonstrated that elders having poor oral health knowledge were more likely to report severely compromised OHRQoL [14]. An intervention to improve American elders’ oral health knowledge has also in turn ameliorated their OHRQoL [15]. Nevertheless, oral health knowledge was found unrelated to OHRQoL in a sample of US adolescent dental patients [16].

Because the literature on the determinants of OHRQoL in adolescents and young adults remained controversial, and to the best of the authors’ knowledge, the relationship between OHRQoL and oral health knowledge of the college population in the US has barely been reported, the purposes of the present study were to fill in the knowledge gap and gain an insight into the self-perceived oral health status of undergraduate students. In particular, the present study aimed to investigate OHRQoL of young adults with a sample of 19-to-24-year-old first-year undergraduates enrolled in the University of Minnesota Twin Cities. A research goal was to examine the relationships between OHRQoL and oral health knowledge as well as demographic factors such as age, gender, and college of study.

## Methods

With the approval from the University of Minnesota Institutional Review Board (IRB) for an IRB exemption (STUDY00014437), this cross-sectional study was carried out during January to March 2022. In October 2021, there were a total of 6,129 first-year undergraduate students enrolled in the University of Minnesota Twin Cities. For confidentiality reasons, four schools/colleges that had fewer than ten freshmen were excluded, resulting in a population size of 6,120 students from eight schools/colleges. The rationale behind the exclusion of the four schools/colleges was that a very small student number at specific schools/colleges could allow people to identify the participants of those small schools/colleges based on their responses to the survey items. The sample size was estimated using an openly available software, Epi Info™ 7.2.3.1 (Centers for Disease Control and Prevention, Atlanta, Georgia, USA, 2019). The following parameters were entered into Epi Info™: (1) a population size of 6,120, (2) an expected frequency of 58.5% based off the percentage for the high level of oral health knowledge in a similar age group [17], (3) an acceptable margin of error of 5%, (4) a design effect of 1.0, and (5) a cluster number of 8 representing eight undergraduate schools. With the aim to achieve a 95% confidence interval, the sample size was estimated to be 352 students. As the estimated sample size was smaller than 500, a minimum response rate of 20% was required to provide confident survey estimates [18]. Thus, a possible negative response rate of 80% was set and this further raised the estimation to 1,760 students. Upon request, the University of Minnesota Office of Institutional Research selected a stratified random sample consisting of 2,200 first-year undergraduate students for the researchers to approach, representing approximately one-third of the population size. To protect students’ privacy, the Office of Institutional Research only provided students’ university email addresses to the researchers. Student names and other personal details were not made available to the researchers.

Students in this sample received an informed consent email from the researchers to explain the research information and advise them not to participate in this study if they were non-English speakers, younger than 18 years of age, considered cognitively impaired, or with fluctuating or diminished capacity to consent. The informed consent email also provided an electronic web address to a self-administered online questionnaire hosted by Qualtrics (Qualtrics International Inc., Provo, Utah, USA, 2022). Upon opening of the online questionnaire, the respondent was presented with an informed consent statement. Consent for the use and interpretation of data was provided upon submitting a response to the online questionnaire as outlined in the informed consent statement.

The survey questionnaire was composed of 39 items, with the first four items seeking informed consent and collecting the data of birth year, gender, and school enrolled. The following 23 items were multiple choice questions with only one correct answer, which were adopted from the Comprehensive Measure of Oral Health Knowledge (CMOHK) questionnaire [9] to assess the level of a respondent’s oral health knowledge. The final questions were four-level Likert items to evaluate the self-perceived frequency of 12 different OHRQoL problems due to the state of their teeth or mouth, which were adopted from the World Health Organization (WHO) Oral Health Questionnaire for Adults. The OHRQoL parameters included (1) difficulty in biting foods, (2) difficulty in chewing foods, (3) difficulty with speech/trouble pronouncing words, (4) dry mouth, (5) felt embarrassed due to appearance of teeth, (6) felt tense because of problems with teeth or mouth, (7) have avoided smiling because of teeth, (8) had sleep that is often interrupted, (9) have taken days off work, (10) difficulty doing usual activities, (11) felt less tolerant of spouse or people who are close to the respondent, and (12) have reduced participation in social activities [19]. Because all relevant items from the CMOHK and WHO Oral Health Questionnaire for Adults were included and no modification of the survey tools was made, a validity test for the questionnaire used in the present study was not indicated.

Data were stored in a Microsoft Excel spreadsheet (Version 2022) and were imported in R (Version 4.2.2). Statistical analysis included descriptive analysis, ordinal logistic regression [20] and Kendall’s tau-b [21]. Ordinal logistic regression models were used to examine the contributions of demographic factors and the level of oral health knowledge to each OHRQoL parameter. A Kendall’s tau-b method was applied for association analysis between each pair of OHRQoL parameters. An association was considered statistically significant if its p-value was less than 0.05. Each OHRQoL parameter (frequency of experiencing an oral health-related problem) was valued as ‘never’, ‘sometimes’, and ‘fairly or very often’. It was treated as an ordinal variable because its values reflected a rank of the frequency but the intervals between the values were not equally spaced. Based on the CMOHK score achieved by the participant, the level of oral health knowledge was categorized as ‘good’ (15–23 scores), ‘fair’ (12–14 scores) and ‘poor’ (0–11 scores) [9], and was treated as an ordinal variable, too. The student’s age was calculated by subtracting the birth year from 2022. The present study did not intentionally exclude 18-year-olds, but there were very few 18-year-olds in this student population and none of them consented to participate. As a result, only those subjects with an age ranging from 19 to 24 years were included in data analysis. Age was also treated as an ordinal variable, with the three categories of ‘19 years’, ‘20 years’, and ‘21 years or older’, due to a right-skewed age distribution which was expected for a survey of first-year undergraduates. Since consent for the use and interpretation of data was not considered obtained, subjects with missing data were excluded from data analysis.

## Results

Four hundred and eighty-five out of the 2,200 randomly selected students consented to participate in the study, and this contributed to a participation rate of 22.1%. A hundred and eleven participants with missing data and another 2 students older than 24 years of age were excluded from data analysis. Thus, a total of 372 students were included in the final sample. The majority of the first-year students in this sample were 19-year-old, female, and enrolled in the College of Liberal Arts (Table 1). The percentage distribution of the participants from each of the eight schools was similar to the proportion of the total first-year students enrolled at each of the eight schools among the University of Minnesota Twin Cities, which indicated that the stratified random sample appropriately represented the student distribution of the schools.

**Table 1:** Frequency distribution of the first-year undergraduate students in a sample of US undergraduate students (n = 372)

	Frequency	%
Age		
19	236	63.4
20	118	31.7
21	16	4.3
22	1	0.3
24	1	0.3
Gender		
Female	246	66.1
Male	104	28.0
Transgender, Non-binary and Other	22	5.9
School		
Biological Sciences	34	9.1
Design	16	4.3
Education and Human Development	32	8.6
Food, Agriculture and Natural Resources Sciences	25	6.7
Liberal Arts	174	46.8
Management	38	10.2
Nursing	8	2.2
Science and Engineering	45	12.1
Total	372	100.0

The CMOHK scores in this sample ranged from 0 to 23. Classified into different groups, two hundred and sixty-five (71.2%) of the respondents demonstrated a ‘good’ level of oral health knowledge, seventy (18.8%) had a ‘fair’ level of oral health knowledge, and 37 (10%) had a ‘poor’ level of oral health knowledge.

**Fig. 1:**
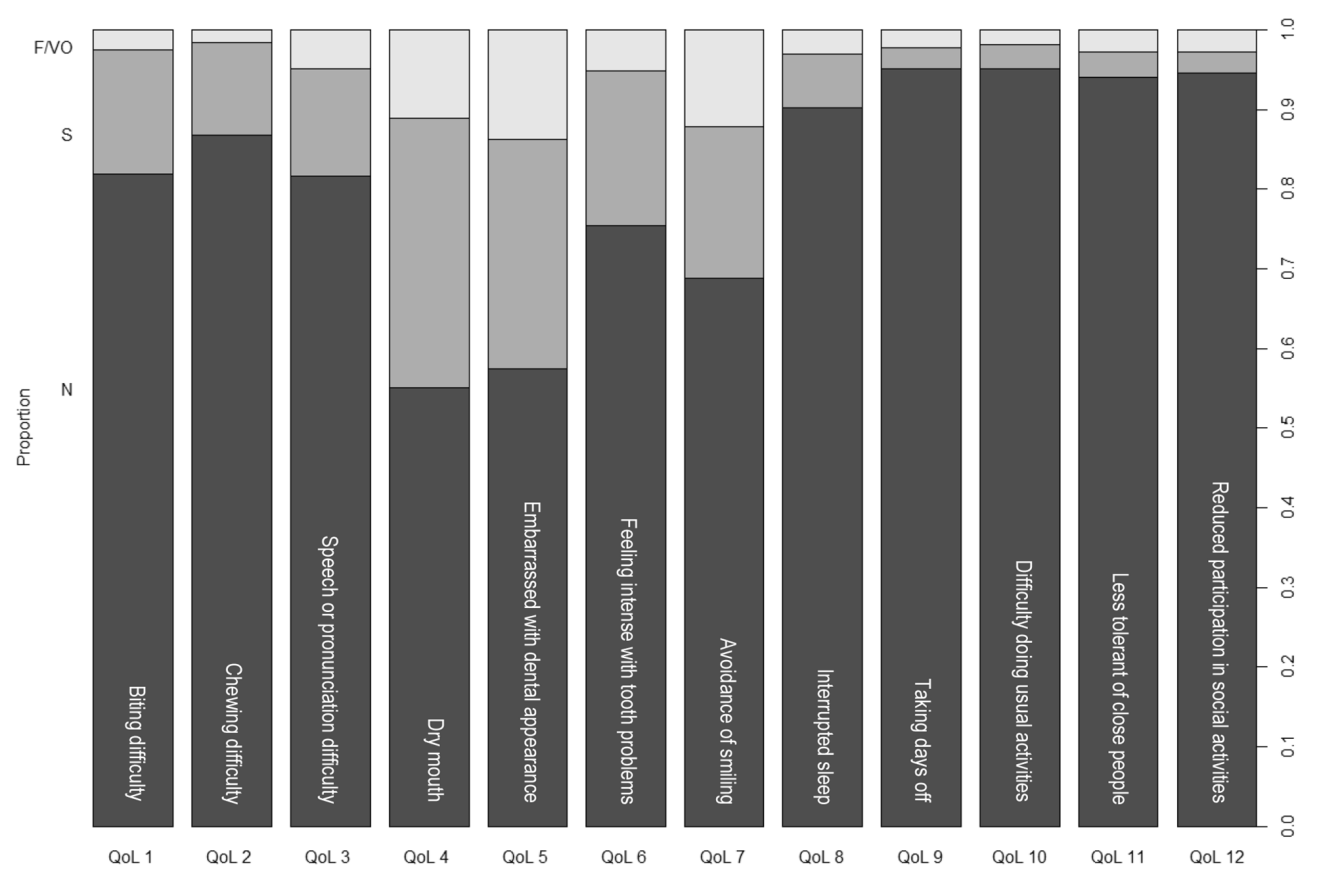
Proportions of different categories for the 12 OHRQoL parameters in a sample of US undergraduate students (n = 372). From dark to light grey: Never (N), Sometimes (S), and Fairly or Very Often (F/VO)

Figure 1 shows the proportions of different categories for the 12 OHRQoL parameters. Notably, there was a higher proportion of respondents experienced problems (either sometimes or more often) with OHRQoL 4 to 7. Dry mouth (OHRQoL 4) and feeling embarrassed due to appearance of teeth (OHRQoL 5) were the most common oral health problems. As high as 45% and 43% of the respondents reported they had experienced dry mouth and felt embarrassed due to appearance of teeth, respectively. Having avoided smiling because of teeth (OHRQoL 7) and having felt tense because of problems with teeth or mouth (OHRQoL 6) were the next two most common problems, with 32% and 25% of the respondents reported the experience of these OHRQoL problems, separately.

The result of ordinal regression for levels of oral health knowledge is summarized in Table 2. Compared to students aged 19, students aged 21 or above were more likely to have poorer oral health knowledge (β = -1.86, *p* = 0.0002). The odds ratio of achieving better oral health knowledge between students aged 21 or above and students aged 19 was 0.16 (95% CI: 0.06–0.42). Also, first-year students enrolled in College of Biological Sciences demonstrated a better level of oral health knowledge (β = 2.35, *p* = 0.0230). The odds of achieving better oral health knowledge for students enrolled in College of Biological Sciences was 10 times that for students enrolled in College of Liberal Arts (95% CI: 2.13–189.08).

**Table 2:** Estimated coefficients (β), and the corresponding odds ratios (OR) and *p*-values, of ordinal regression model for CMOHK against age, gender and school enrolled in a sample of US undergraduate students (n = 372). Threshold coefficients for “Good”, and the parameters of the baseline level for all explanatory variables were set to zero. *P*-values less than 0.05 are indicated by an asterisk

	Estimate (β)	Standard Error	OR	*p*-value
Threshold coefficients				
Poor	-2.63	0.26	0.07	< 0.0001*
Fair	-1.22	0.22	0.30	< 0.0001*
Good	---		---	---
Age				
19	---		---	---
20	-0.35	0.25	0.71	0.1681
21 or above	-1.86	0.51	0.16	0.0002*
Gender				
Female	---		---	---
Male	-0.35	0.26	0.70	0.1832
Transgender, Non-binary and Others	0.42	0.58	1.52	0.4757
School				
Biological Sciences	2.35	1.03	10.46	0.0230*
Design	-0.76	0.54	0.47	0.1553
Education and Human Development	-0.59	0.40	0.55	0.1425
Food, Agriculture and Natural Resources Sciences	0.48	0.52	1.62	0.3532
Liberal Arts	---		---	---
Management	-0.49	0.37	0.61	0.1839
Nursing	-0.43	0.72	0.65	0.5479
Science and Engineering	0.05	0.38	1.05	0.8990

Table 3 shows the estimated coefficients for the ordinal regression models for the 12 OHRQoL parameters. Age had a significant association with OHRQoL parameter 6. Compared with the students aged 19, those who were age 21 or above had a higher chance of experiencing tense feeling because of problems with teeth or mouth more frequently (β = 1.12, *p* = 0.0283). A significant association between gender and OHRQoL parameters 3, 6 and 12 were found. Compared to female students, male students experienced speech or pronunciation difficulty less frequently (β = -0.75, *p* = 0.0333). In addition, respondents of transgender/non-binary/other gender were more likely to experience tense feeling because of problems with teeth or mouth (β = 0.91, *p* = 0.0471) and reduce participation in social activities due to oral health issues (β = 1.61, *p* = 0.0281) than females.

**Table 3:** Estimated coefficients (β), standard errors (SE), and *p*-values (p) of the ordinal regression models for the OHRQoL parameters in a sample of US undergraduate students (n = 372). Threshold coefficients for the last level, and the parameters of the baseline level for all explanatory variables were set to zero. *P*-values less than 0.05 are indicated by an asterisk

OHRQoL parameters		1. Biting difficulty	2. Chewing difficulty	3. Speech or pronunciation difficulty	4. Dry mouth	5. Embarrassed with dental appearance	6. Feeling intense with tooth problems
Threshold coefficients							
Never	β SE *p*	0.85 0.42 0.0435*	1.32 0.46 0.0004*	0.30 0.41 0.4696	-0.24 0.36 0.5072	0.12 0.35 0.7312	0.83 0.40 0.0378*
Sometimes	β SE *p*	3.05 0.52 < 0.0001*	3.57 0.59 <0.0001*	1.81 0.45 0.0001*	1.65 0.37 <0.0001*	1.67 0.37 <0.0001*	2.68 0.45 <0.0001*
Fairly or very often		---	---	---	---	---	---
Age							
19		---	---	---	---	---	---
20	β SE *p*	0.13 0.29 0.6601	0.33 0.33 0.3201	-0.07 0.30 0.8097	0.03 0.22 0.8760	0.23 0.22 0.2871	-0.14 0.28 0.6116
21 or above	β SE *p*	-0.31 0.69 0.6582	0.74 0.60 0.2182	0.16 0.61 0.7882	0.10 0.52 0.8780	0.55 0.50 0.2739	1.12 0.51 0.0283*
Gender							
Female		---	---	---	---	---	---
Male	β SE *p*	-0.31 0.32 0.3331	-0.15 0.35 0.6775	-0.75 0.35 0.0333*	-0.16 0.24 0.5065	-0.09 0.24 0.6924	-0.24 0.29 0.4071
Transgender, Non-binary and Others	β SE *p*	-0.80 0.76 0.2944	-1.05 1.04 0.3169	0.01 0.58 0.9789	-0.01 0.43 0.9887	0.30 0.42 0.4772	0.91 0.46 0.0471*
Level of Oral Health Knowledge							
Poor		---	---	---	---	---	---
Fair	β SE *p*	-0.86 0.52 0.0964	-0.46 0.53 0.3815	-1.28 0.50 0.0112*	-0.51 0.40 0.2055	-0.30 0.40 (0.4566	-0.89 0.49 0.0718*
Good	β SE *p*	-0.59 0.42 0.1601	-0.82 0.46 0.0731	-1.12 0.41 0.0006*	-0.44 0.36 0.2119	-0.30 0.35 0.3888	-0.24 0.40 0.5532
OHRQoL parameters		7. Avoidance of smiling	8. Interrupted sleep	9. Taking days off	10. Difficulty doing usual activities	11. Less tolerant of close people	12. Reduced participation in social activities
Threshold coefficients							
Never	β SE *p*	0.65 0.38 0.0917	1.16 0.48 0.0154*	2.08 0.61 0.0007*	1.76 0.59 0.0027*	1.87 0.61 0.0023*	1.98 0.59 0.0008*
Sometimes	β SE *p*	1.84 0.40 <0.0001*	2.45 0.54 <0.0001*	2.96 0.66 <0.0001*	2.81 0.65 <0.0001*	2.71 0.65 <0.0001*	2.73 0.63 <0.0001*
Fairly or very often		---	---	---	---	---	---
Age							
19		---	---	---	---	---	---
20	β SE *p*	0.12 0.24 0.6191	-0.25 0.41 0.5454	-0.15 0.58 0.7914	-0.10 0.60 0.8740	-0.34 0.54 0.5363	-0.20 0.56 0.7232
21 or above	β SE *p*	0.80 0.47 0.0902	0.11 0.72 0.8769	0.75 0.79 0.3404	0.97 0.76 0.2008	0.90 0.78 0.2483	0.71 0.78 0.3639
Gender							
Female		---	---	---	---	---	---
Male	β SE *p*	-0.01 0.25 0.9612	-0.06 0.41 0.8810	0.87 0.53 0.1011	0.49 0.55 0.3714	-0.29 0.55 0.5953	0.38 0.52 0.4709
Others	β SE *p*	-0.63 0.57 0.2716	0.59 0.68 0.3853	1.48 0.85 0.0825	1.56 0.87 0.0724	1.18 0.70 0.0913	1.61 0.73 0.0281*
Level of Oral Health Knowledge							
Poor		---	---	---	---	---	---
Fair	β SE *p*	-0.15 0.43 0.7343	-0.56 0.55 0.3053	-1.52 0.78 0.0526	-1.38 0.70 0.0501	-0.90 0.75 0.2332	-0.83 0.69 0.2300
Good	β SE *p*	-0.24 0.38 0.5279	-1.43 0.50 0.0040*	-1.71 0.62 0.0054*	-2.37 0.64 0.0002*	-1.11 0.63 0.0771	-1.65 0.62 0.0078*

--- used as the baseline reference in ordinal regression models

The level of oral health knowledge was related to OHRQoL parameters 3, 8, 9, 10 and 12. Compared to students with a poor level of oral health knowledge, those who had a fair or good level of knowledge experienced difficulty with speech or trouble pronouncing words less often (fair: β = -1.28, *p* = 0.0112; good: β = -1.12, *p* = 0.0006). Also, students having a good level of knowledge were less likely to experience interruption during sleep (β = -1.43, *p* = 0.0040), less likely to take days off work due to oral health problems (β = -1.71, *p* = 0.0054), less likely to experience difficulty doing usual activities (β = -2.37, *p* = 0.0002), and less likely to reduce participation in social activities (β = -1.65, *p* = 0.0078), when compared to respondents with a poor level of oral health knowledge.

Table 4 summarizes the Kendall’s tau-b measures and the corresponding *p*-values for each pair of the 12 OHRQoL parameters. All OHRQoL parameters were positively correlated, with a range between 0.09 and 0.67 and a median of 0.22. All measures were statistically significant, except the one between OHRQoL parameters 3 and 5 (*p* = 0.0546). The correlations between OHRQoL parameters 1 and 2, 5 and 7, 8 and 9, 9 and 10, 9 and 12, 10 and 11, 10 and 12, as well as 11 and 12, are viewed as ‘strong’ associations (Kendall’s tau-b ≥ 0.5). Most other OHRQoL parameters show ‘moderate’ correlations between each other (0.2 ≤ Kendall’s tau-b < 0.5).

**Table 4:** Kendall’s tau-b measures (*p*-values in parentheses) for each pair of the 12 OHRQoL parameters in a sample of US undergraduate students (n = 372)

OHRQoL Parameter	1. Biting difficulty	2. Chewing difficulty	3. Speech or pronunciation difficulty	4. Dry mouth	5. Embarrassed with dental appearance	6. Feeling intense with tooth problems	7. Avoidance of smiling	8. Interrupted sleep	9. Taking days off	10. Difficulty doing usual activities	11. Less tolerant of close people	12. Reduced participation in social activities
1. Biting difficulty	1.00	0.57 (< 0.0001)	0.22 (< 0.0001)	0.23 (< 0.0001)	0.28 (< 0.0001)	0.35 (< 0.0001)	0.20 (0.0001)	0.28 (< 0.0001)	0.21 (< 0.0001)	0.17 (0.001)	0.21 (< 0.0001)	0.20 (0.0001)
2. Chewing difficulty		1.00	0.17 (0.0007)	0.16 (0.0012)	0.20 (0.0001)	0.18 (0.0004)	0.18 (0.0004)	0.36 (< 0.0001)	0.29 (< 0.0001)	0.21 (< 0.0001)	0.25 (< 0.0001)	0.23 (< 0.0001)
3. Speech or pronunciation difficulty			1.00	0.20 (< 0.0001)	0.09 (0.0546)	0.20 (0.0001)	0.13 (0.0075)	0.19 (0.0002)	0.23 (< 0.0001)	0.20 (0.0001)	0.16 (0.0014)	0.27 (< 0.0001)
4. Dry mouth				1.00	0.20 (< 0.0001)	0.19 (0.0001)	0.19 (0.0001)	0.19 (0.0001)	0.14 (0.0054)	0.15 (0.0028)	0.16 (0.0010)	0.23 (< 0.0001)
5. Embarrassed with dental appearance					1.00	0.37 (< 0.0001)	0.59 (< 0.0001)	0.24 (< 0.0001)	0.20 (< 0.0001)	0.13 (0.0067)	0.22 (< 0.0001)	0.28 (< 0.0001)
6. Feeling intense with tooth problems						1.00	0.31 (< 0.0001)	0.30 (< 0.0001)	0.34 (< 0.0001)	0.24 (< 0.0001)	0.26 (< 0.0001)	0.31 (< 0.0001)
7. Avoidance of smiling							1.00	0.20 (< 0.0001)	0.21 (< 0.0001)	0.13 (0.0078)	0.18 (0.0003)	0.27 (< 0.0001)
8. Interrupted sleep								1.00	0.57 (< 0.0001)	0.45 (< 0.0001)	0.36 (< 0.0001)	0.49 (< 0.0001)
9. Taking days off									1.00	0.54 (< 0.0001)	0.44 (< 0.0001)	0.55 (< 0.0001)
10. Difficulty doing usual activities										1.00	0.53 (< 0.0001)	0.56 (< 0.0001)
11. Less tolerant of close people											1.00	0.67 (< 0.0001)
12. Reduced participation in social activities												1.00

## Discussion

The present study has demonstrated a preventive potential of better oral health knowledge on specific OHRQoL problems such as speech or pronunciation difficulty, interrupted sleep, taking days off, difficulty doing usual activities, and reduced participation in social activities. This agreed with Márquez-Arrico et al. who have also reported a similar effect on adult dental patients’ difficulty with speech or pronunciation, taking days off, and difficulty doing usual activities, even though their work did not identify a relationship between oral health knowledge and interrupted sleep as well as reduced participation in social activities [17]. Interestingly, the proportions of young adults experiencing interrupted sleep and reduced participation in social activities, as reported in the present study, were lower than the proportions of older adults experiencing the same OHRQoL problems, as reported by Márquez-Arrico et al. Because older adults sustained insomnia often and participated in social activities less frequently [22], they may not consider those issues as oral health-related. And consequently, the associations between oral health knowledge and oral health-related sleep disruption/social inactivity were significant in young adults but not in older individuals.

Because a better level of oral health knowledge was associated with a reduced frequency of unhealthy oral health behavior [10] and an excellent level of oral health [23], those who had better oral health knowledge would consequently also report better OHRQoL, as demonstrated in the present and past studies [12, 14, 17]. Nevertheless, why was oral health knowledge relevant to some but not all OHRQoL issues in this sample? In Table 4, except for speech or pronunciation difficulty, the other four OHRQoL parameters (interrupted sleep, taking days off, difficulty doing usual activities, and reduced participation in social activities) showed stronger correlations among each other, with a Kendall’s tau-b measure ranging from 0.45 to 0.57. Those four OHRQoL parameters had lower correlations with most other OHRQoL problems. Based on the 4-dimensional OHRQoL structure [11], speech or pronunciation difficulty can be classified in the Dimension of Oral Function; interrupted sleep can be partially classified in the Dimension of Orofacial Pain and partially in the Dimension of Psychosocial Impact; and taking days off, difficulty doing usual activities, and reduced participation in social activities are also classified in the Dimension of Psychosocial Impact. Thus, oral health knowledge was mainly associated with the Dimension of Psychosocial Impact in young adults’ OHRQoL. This concurred with Gil-Lacruz et al. who have demonstrated that the effect of education on young adults’ health-related quality of life was most notable in the mental health dimension [24]. On the other hand, oral health knowledge was not related to patients’ history of seeking urgent care for dental problems [25]. As the most common conditions requiring urgent dental care included infection, pain, trauma and restoration fractures which are all pain or function-related issues [26], it is understandable that oral health knowledge is a strong determinant for the psychosocial dimension but weaker for other dimensions of OHRQoL.

The percentage of dry mouth subjects identified in the present study was surprisingly higher than the prevalence observed in similar age groups of other countries [27, 28]. Although a previous study has found cigarette smoking and use of electronic cigarettes as determinants of dry mouth among college students [28], the prevalence of past 30-day smoking in American undergraduates was only a half of the dry mouth rate reported in the present study [29]. This suggested that dry mouth is a developing public health challenge in young adults and indicated the relevance to investigate the etiology of dry mouth in this population. The present study also reported a high percentage of 19-to-24-year-olds feeling dissatisfied with their own dental appearances, which agreed with past studies describing adolescents and young adults’ self-perceived problems in tooth alignments and colors/shades [30, 31]. With a quarter of American women having unmet dental care needs due to high costs, uncovered dental procedures, or impractical belief [32], and two thirds of American adults exhibiting untreated malocclusions [33], no wonder so many college freshmen felt uneasy about their dental appearances. Because dry mouth and feeling embarrassed about dental appearance were not associated with levels of oral health knowledge, improving young adults’ access to dental care may be more effective than developing health promotion strategies for these OHRQoL problems.

Despite a reverse association between age and level of oral health knowledge was reported in the present study, it should also be noted that the proportion of first-year undergraduate students aged 21 years or above was quite small. Two previous studies, one using a sample of Saudi Arabic school teachers [34] and the other investigating American dental patients [35], have also suggested a decrease of oral health knowledge following the increase of age. Loss of motivation to acquire or memorize oral health knowledge is possible for elders, but the participants of the present study were young adults who were unlikely to lose motivation in their early twenties. Thus, other age-related reasons could result in the reverse association. For instance, students learned oral health knowledge at middle school or high school and gradually forgot about it as time went by. To figure out the reason why older students showed a lower level of oral health knowledge, future investigation including sophomore, junior and senior students is indicated.

Of further note, first-year undergraduate students in the College of Biological Sciences showed a higher level of oral health knowledge than those enrolled in the College of Liberal Arts. If the students were selected from the sophomore, junior or senior years, the difference could result from the health-related content learned in the College of Biological Sciences. Nevertheless, all participants were in their first year of study. The learning experience in their first semester at the university would be less likely to form 10 times odds between the two colleges. Hence, the health-related interest that Biological Sciences students have gradually developed since an earlier age may be the reason for their robust oral health knowledge demonstrated in this survey.

Comparison of our findings with other studies in relation to OHRQoL parameters was challenging, since the tools used to assess OHRQoL varied among studies. The WHO Oral Health Questionnaire for Adults were adopted by Márquez-Arrico et al. [17], Jahangiry et al. [36] and the present study, while some other studies opted to use the Oral Health Impact Profile-14 (OHIP-14) [12, 14, 15], Oral Health Impact Profile for Edentulous People (OHIP-EDENT) [37], Child Perceptions Questionnaire (CPQ) [38], Child Oral Impact on Daily Performances (C-OIDP) [39], or Control, Autonomy, Self-realization and Pleasure-12 (CASP-12) [40]. While OHIP-14, OHIP-EDENT, and CPQ used a 5-point Likert scale to evaluate the frequency of OHRQoL problems, C-OIDP, CASP-12 and the WHO Oral Health Questionnaire for Adults used a 4-point Likert scale for the same purpose. The OHRQoL tools using 4-point and 5-point Likert scales are all validated and commonly used. Also, the present study used CMOHK to evaluate undergraduate students’ oral health knowledge and this tool has been used by some other researchers [9, 14, 17, 25]. Kanupuru et al. have used Rapid Estimation of Adult Literacy in Dentistry (REALD-99) [6], and Zheng et al. have developed their own tool to evaluate Chinese college students’ oral health knowledge [12]. Although using the WHO Oral Health Questionnaire for Adults and CMOHK to examine the relationship between OHRQoL and oral health knowledge in first-year undergraduate students created a unique and novel position for the present study, this also became one of the study’s limitations.

Moreover, the majority of the undergraduate students enrolled at the University of Minnesota were from the State of Minnesota. Additionally, almost half of the participants were enrolled in the College of Liberal Arts. Although the percentage distribution of participants from each school/college was quite similar to that of the total freshmen enrolled in each school/college, it would be adventurous to make a generalization applying to the young adult populations of other states and countries, or with different educational backgrounds. Further, the educational environment, student expectation and/or learning experience may differ among schools and colleges, and these in turn may have influenced the research outcomes reported in this paper. Nevertheless, the present study collected the data from first-year undergraduate students at the time when the second semester of their freshman year just started. The timing of data collection would reduce the influence from the educational environment, student expectation and/or learning experience on the students’ responses.

With a participation rate of 22.1%, and a female-to-male ratio of 2:1 in the respondents, the potential presence of sampling bias was another limitation of the present study. To improve the generalizability of the study, taking a multicenter approach and providing incentives to participants can be considered in future investigations. Also, due to the challenge to engage the same students in repeating tests at different times, a test-retest reliability was not measured in the present study. To strengthen the confidence in the consistency of the research method used, improvement of student engagement in survey participation and evaluation of a test-retest reliability are indicated.

Another limitation of the present study lies in the fact that we could not investigate the effect of missing data to the results reported. Due to ethical concerns, participants with missing data, which comprised of approximately one-quarter of the data received, were considered as non-consensual for the use of data. Therefore, the data received were completely omitted during the analysis. It is acknowledged that estimates from subjects with complete data only could potentially be biased.

Currently there are no low-cost dental services specially designed for university students. To improve students’ OHRQoL, universities can develop a strategic plan to provide affordable dental services to students and incorporate oral health knowledge into the curriculum of general education classes. These can be supported by a recent study showing students’ interest in acquiring oral health knowledge at university and using campus-based dental services [41]. Further, an interprofessional educational program to let dental and nursing students collaboratively provide oral health education and oral hygiene instruction to long-term care residents has resulted in an increase of the elders’ oral health knowledge [42]. Their successful approach indicates a feasible direction to include dental, health professional and education professional students in the university-wide and/or inter-institutional oral health education scheme. This may help to narrow the gap of oral health knowledge among the students at different colleges and schools, as presented by the present study. Also, as dry mouth is becoming prevalent in this population, raising students’ awareness of dry mouth and healthy hydration habits is also indicated. Further investigations on causality between oral health knowledge and OHRQoL problems are required as well.

## Conclusions

The present study has demonstrated better oral health knowledge as a protective factor for selected OHRQoL problems in first-year undergraduate students. Dry mouth and feeling embarrassed with dental appearance were the most prevalent OHRQoL issues in this population, and neither was related to the level of oral health knowledge. To improve OHRQoL at universities, this paper recommends to incorporate oral health knowledge into the curriculum of general education classes, include students in university-wide interprofessional schemes of oral health education, provide affordable dental services to students, and raise awareness of dry mouth and healthy hydration habits. Future investigation on causality between oral health knowledge and OHRQoL problems is indicated.

## Data Availability

The datasets used and/or analyzed in the present study are available from the corresponding author on reasonable request.

## List of abbreviations

CASP-12: Control, Autonomy, Self-realization and Pleasure-12.
CMOHK: Comprehensive Measure of Oral Health Knowledge.
C-OIDP: Child Oral Impact on Daily Performances.
CPQ: Child Perceptions Questionnaire.
IRB: Institutional Review Board.
OHIP-14: Oral Health Impact Profile-14.
OHIP-EDENT: Oral Health Impact Profile for Edentulous People.
OHRQoL: Oral health-related quality of life.
REALD-99: Rapid Estimation of Adult Literacy in Dentistry.
US: United States.
WHO: World Health Organization.
